# What factors influencing surgical nurses’ competence in implementing person-centered care in the perioperative period?: A cross-sectional study

**DOI:** 10.1097/MD.0000000000040035

**Published:** 2024-10-11

**Authors:** Lu Shen, Dongju Xu, Xiumei Zhang

**Affiliations:** a Department of Cardiovascular Medicine, The First Affiliated Hospital of Anhui Medical University, Hefei, Anhui, China; b Department of Nursing, The First Affiliated Hospital of Anhui Medical University, Hefei, Anhui, China.

**Keywords:** competence, empathy, influencing factors, job burnout, person-centered care, surgical nurses

## Abstract

Strengthening the capability of clinical surgical nurses to provide person-centered care (PCC) requires a thorough analysis of several related factors. This study used a descriptive cross-sectional design to investigate the factors that influence surgical nurses’ perioperative competency in the performance of PCC in Anhui, China. A convenience sampling was implemented to administer a questionnaire survey to 437 nurses with more than 1 year of experience working in surgical wards. A web-based cross-sectional questionnaire evaluated the participant’s general sociological information, empathy, job burnout, and nursing competence. The questionnaire were designed and published through the online platform Questionnaire Stars, and the link was distributed through the Internet and WeChat media by the nursing department of their hospital. Data were collected from June to September 2023 and processed by descriptive, correlation, and stepwise multiple regression analyses. Participants with high levels of empathy (*β* **= **0.502, *P* < .001), low levels of job burnout (*β* **= −**0.288, *P* < .001), humanistic nursing training (*β* **= **0.167, *P* < .001), and personnel agency (*β* **= **0.083, *P* < .001) showed better PCC competence. The regression model analysis showed that 4 influencing factors explained 59.2% of the variance (*F* = 158.930, *P* < .001, adjusted *R*^2^ = 0.592). These findings suggest that steps should be taken to strengthen the key factors, such as improving empathy, minimizing job burnout, offering more humanities training, and enhancing personalities, to reinforce PCC competency.

## 1. Introduction

Over the last few decades, significant advances have been made in the understanding of the qualities, requirements, and processes of surgical nursing. Surgical nurses worldwide are working towards ensuring patient safety, and needs and promoting rapid recovery, which is the most challenging aspect of clinical nursing.^[1,2]^ In this circumstance, and as an inevitable consequence of a paradigm shift in medicine, there is a significant interest in person-centered care. Person-centered care evolves as an unavoidable outcome and a critical component of the growth of the medical humanities. Florence Nightingale proposed that the most outstanding quality of nursing and medicine is the focus on the patient than the disease. The concepts of “person-centered care” and “patient-centered care” are often cited interchangeably in the medical profession, indicating that the patient should be seen as the focus of nursing.^[3]^ In this study, we propose a broad concept of “person-centered care (PCC).” McCormack and McCance mid-range framework for PCC focuses on 4 components of clinical practice: conditions, environment, person-centered procedure, and consequences.^[4,5]^ Consequently, PCC is more similar to caring for patients from their perspective, including respecting, listening to, and promoting their preferences with the aim of achieving health promotion and illness prevention goals.^[6]^

Although surgical safety has been dramatically improved, the risks associated with the perioperative period, postoperative rehabilitation, and prevention of complications affect the patient’s clinical benefits and overall quality of life.^[7,8]^ Previous reports have demonstrated a marked association between PCC and the following outcomes. Firstly, patients who received PCC achieved better health status, including reduced wound pain, enhanced mobility and greater satisfaction with care.^[9]^ In addition, it can improve nurses’ career satisfaction, and reduce burnout and pressure, thus improving the quality of care.^[10]^ Therefore, PCC has emerged as a fundamental aspect of clinical practice that requires ongoing development, given its immense value for both patients and caregivers.

Practitioners must possess the necessary humanistic competence, because PCC is a crucial tool for patients to participate in decision-making. Most global studies on humanistic competence have focused on South Korea and Japan and continue to develop their theory and practice.^[11–13]^ Conversely, China has a vast population base, and its healthcare approach is mostly disease-oriented and focuses on medical condition problems. Owing to these challenges in adopting humanistic care, studies on these competencies are uncommon. PCC competencies among nursing students and clinical interns were measured and improved to enable undergraduate nurses to develop positive and effective humanistic practices before work.^[11–13]^ Earlier research has identified empathy,^[14]^ professional nursing competence,^[12]^ clinical learning environment,^[15]^ nursing professional values,^[16]^ and thinking strategies^[17]^ as relevant factors influencing nursing competence. In addition, some cross-sectional studies have also compared the influence of basic demographics, such as gender, age, and academic level, on the realization of PCC.^[18,19]^ Those studies have used exploratory methods to examine specific factors on humanistic competence; however, as it is a comprehensive concept, it is not easy to account for them with a single variable.

Therefore, the objective of this study was to highlight the factors that influence PCC among surgical nurses during the perioperative period in Anhui, China, and to encourage the development of a program to increase nurses’ knowledge and competence, and provide relevant data and empirical support. We hypothesized that surgical nurses have a high level of PCC competence and that personal, environmental, and learning factors are the relevant factors influencing humanistic abilities.

## 2. Methods

### 2.1. Study design

The study was a descriptive, cross-sectional survey, and followed to the STROBE guideline for cross-sectional studies.

### 2.2. Participants

The participants recruited from June to September 2023 were surgical in-service nurses from 4 tertiary hospitals in Hefei, Anhui, China. All participants answered the questionnaire issued by the nursing department of their hospital through the online platform. The inclusion criteria were that clinical and surgical nurses must obtain professional nurse qualification certificates in mainland China and have more than 1 year of working experience in clinical surgical departments. Nurses who are sick, on rotation or on vacation are excluded. This study was approved by the Medical Research Ethics Committee of the First Affiliated Hospital of Anhui Medical University (Approval No. PJ2023-05-39), and online informed consent was obtained from all participants in accordance with the tenets of the Declaration of Helsinki. Considering that the sample size should be 5 to 10 times the number of variables and the release rate is 10%, this study had at least 77 to 154 research data. A total of 452 questionnaires were distributed in this study, and data with inappropriate response time (n = 9), strong regularity (n = 4), and obvious contradictions (n = 2) were deleted. Four hundred thirty-seven valid questionnaires were recovered, with an effective return rate of 96.7%.

### 2.3. Data collection

A web-based cross-sectional questionnaire evaluated the participant’s empathy, burnout and PPC nursing competence. The written informed consent for the study, the survey description, and the questionnaire content were designed and published through the online platform Questionnaire Stars, and the link to the questionnaire was distributed through the Internet and WeChat media. Before performing the survey, participants were all provided with informed consent. Participants can answer the questionnaire at any time they wanted to ensure the accuracy of the results.

### 2.4. Measurements

#### 2.4.1. General nurses information questionnaire

Participants completed a general nurse’s information questionnaire first. The investigation items included age, sex, level of education, marital status, department, working years, professional title, position, family situation, employment status, whether they come from an one-child family or have participated in a humanistic nursing education.

#### 2.4.2. The Person-Centered Perioperative Nursing Scale (PCPON)

The scale used in this study was initially developed by Korean scholars Shin and Kang in 2019, to assess perioperative nursing competence of registered professional nurses.^[20]^ The scale selected in the study was revised and validate in Chinese by a previous research.^[21]^ It includes 5 dimensions and 20 items on compassionate interaction, respect, comfort, sharing information, and expertise. It is measured on a 5-point Likert scale (1 = never, 5 = always) and the total score ranges from 20 to 100. The scale’s content was brief, and nurses chose appropriate options based on their current scenario, which could be completed in as little as 5 minutes. A higher score indicates a higher level of perioperative nursing competence. In our study, Cronbach alpha coefficient for real competence was 0.92 in the early research stage and was 0.93 in our study.

#### 2.4.3. The Jefferson Scale of Empathy-Health Professionals (JSE-HP)

Hojat and his research team developed the Jefferson Scale of Empathy (JSE) in 2001, with a version specifically designed to measure empathy in healthcare professionals (JSE-HP). We chose the JSE-HP, first translated into Chinese by Chinese scholar An Xiuqin.^[22]^ The scale has been proved to have good reliability and validity, and consisted of 20 items, each answered on a 7-point Likert scale varying from 1 (*strongly disagree*) to 7 (*strongly agree*), belonging to 3 dimensions (perspective-taking, perspective-taking, and walking in the patient’s shoes). The higher the score indicates more empathy. The Cronbach *α* of the JSE-HP was 0.75 and 0.94 in this study.

#### 2.4.4. The Maslach Burnout Inventory-General Survey (MBI-GS)

The MBI-GS was used to assess job burnout among nurses. The Chinese version of the MBI-GS, developed by Li Chaoping and Shi Kan performed,^[23]^ consists of 15 items and 3 dimensions: emotional exhaustion (5 items), deindividuation (4 items), and personal accomplishment (6 items). By Li Yongxin assessment criteria for the Chinese version of MBI-GS, each item adopts the 7-step Likert scoring method, and “*never*” to “*every day*” are counted as 1 to 7 points for each dimension.^[24]^ The score was calculated by summing the corresponding items, in which the dimensions of personal accomplishment were scored inversely. The threshold values for the 3 dimensions were 25, 11, and 16 points. High levels of burnout are predicted by high scores on emotional exhaustion and deindividuation and low scores on personal accomplishment. The survey has been reliable and validated, with Cronbach alpha real competence coefficient of 0.74,^[23]^ and 0.82 in this research.

### 2.5. Statistical analyses

All statistical analyses were performed using the IBM SPSS Statistics ver. 20.0 software (IBM Corp., Armonk, NK). Quantitative variables are expressed as mean, standard deviation, or frequency (%) for all outputs. Independent *t* test and the nonparametric test were adopted to draw out the different aspects of general sociological characteristics. Pearson correlation coefficients were calculated to identify the correlations between the study variables. The influence of socio-demographic factors, empathy, and job burnout on PCC was assessed using multivariable hierarchical linear regression analysis. Statistically significance was defined as *P* < .05.

## 3. Results

### 3.1. Levels of PCC competence by demographical characteristics

The differences in perioperative PCC competence according to the categorization of participants’ demographic characteristics are summarized in Table 1. Most of the patients were aged ≤40 years (95.1%), and 97.3% were females. About 72.8% of the participants were signed contract nurses (labor dispatching), 85.1% had bachelor’s degrees, and 62.2% were married. In addition, 85.1% of the participants reported receiving humanistic nursing training for over 1 hour. No statistically significant differences were found in the mean PCC competence for gender (*t* = 0.100, *P* = .921) or whether it belonged to the one-child family (*t* = 0.319, *P* = .750).

**Table 1:** Levels of PCC competence according to demographic characteristics (n = 437).

Characteristics	Categories	N	Value %	Mean (SD)	F/t	*P*-value
Gender					0.100†	.921
	Male	12	2.7	82.83 (8.82)		
	Female	425	97.3	82.55 (9.78)		
Age (years)					45.681‡	<.001
	≤29	227	51.9	79.83 (9.84)		
	30~	189	43.2	84.95 (8.76)		
	40~	17	3.9	89.47 (7.47)		
	≥50	4	0.9	94.75 (3.50)		
Marital status					16.547‡	<.001
	Unmarried	155	35.5	80.09 (9.89)		
	Married	272	62.2	83.96 (9.45)		
	Divorce	10	2.3	82.70 (8.76)		
Education level					9.079‡	.011
	Diploma	55	12.6	80.58 (11.65)		
	Bachelor	372	85.1	82.62 (9.38)		
	≥Master	10	2.3	91.00 (7.59)		
Professional title					33.050‡	<.001
	Nurse	96	22.0	79.74 (9.93)		
	Senior nurse	177	40.5	81.23 (10.05)		
	Supervisor nurse	158	36.2	85.30 (8.41)		
	Associate professor of nursing	6	1.4	94.50 (2.74)		
Duration working as a nurse (years)					38.481‡	<.001
	1~	150	34.8	79.36 (9.74)		
	6~	160	36.6	82.54 (9.53)		
	11~	109	24.9	85.86 (8.80)		
	≥21	18	4.1	89.28 (7.43)		
One-child Family					0.319†	.750
	Yes	79	18.1	82.87 (10.44)		
	No	358	81.9	82.49 (9.60)		
Place of residence					−4.475†	<.001
	Rural area	181	41.1	80.13 (9.16)		
	City or town	256	58.6	84.27 (9.80)		
Employment status					9.955‡	.007
	Labor dispatching	318	72.8	81.72 (9.70)		
	Personnel agency	110	25.2	84.49 (9.70)		
	Authorized personnel	9	2.1	88.33 (7.09)		
Nursing humanistic training (≥1 hour)					8.375†	<.001
	Yes	372	85.1	84.07 (9.36)		
	No	65	14.9	73.88 (7.08)		

PCC = person-centered care, SD = standard deviation.

† *t* test.

‡ Kruskal–Walllis test.

### 3.2. Participant’s PCC competence, empathy, and job burnout

The results of the analysis of the subjects’ PCC competence, empathy, and job burnout are listed in Table 2.

**Table 2:** Descriptive statistics on PCC competence, empathy and job burnout (n = 437).

Variable	Subdimensions	Item	Mean (SD)
PCPON		20	82.56 (9.75)
	Compassionate interaction	6	24.25 (3.13)
	Respect	4	16.90 (2.08)
	Comfort	4	16.76 (2.32)
	Sharing information	3	12.40 (2.08)
	Expertise	3	12.25 (1.95)
JSP-HE		20	114.53 (15.75)
	Perspective-taking	10	59.85 (9.10)
	Compassionate care	7	38.15 (5.90)
	Walking in the patient’s shoes	3	16.53 (2.96)
MBI-GS		15	46.84 (12.49)
	Emotional exhaustion	5	17.13 (5.44)
	Depersonalization	4	13.60 (5.00)
	Personal accomplishment	6	16.11 (6.82)

JSP-HE = Jefferson Scale of Empathy-Health Professionals, MBI-GS = Maslach Burnout Inventory-General Survey, PCC = person-centered care, PCPON = the Person-Centered Perioperative Nursing Scale, SD = standard deviation.

### 3.3. Correlations among the study variables

Table 3 presents the analysis results of the PCPON and JSP-HE, revealing a strong positive correlation (*R* = 0.700, *P* < .001) between PCC competence and empathy. Also, it represents the results of the PCPON and MBI-GS, demonstrating the negative correlation (*r* = −0.583, *P* < .001) between PCC competence and job burnout.

**Table 3:** Correlations among PCC competence, empathy, and job burnout (n = 437).

Variable	Subdimensions	Compassionate interaction	Respect	Comfort	Sharing information	Expertise	PCPON
JSP-HE		.598**	.579**	.603**	.609**	.551**	.700**
	Perspective-taking	.527**	.523**	.523**	.544**	.496**	.621**
	Compassionate care	.552**	.536**	.577**	.562**	.495**	.648**
	Walking in the patient’s shoes	.461**	.408**	.450**	.447**	.421**	.522**
MBI-GS		−.462**	−.469**	−.510**	−.529**	−.499**	−.583**
	Emotional exhaustion	−.415**	−.396**	−.468**	−.503**	−.459**	−.529**
	Depersonalization	−.350**	−.336**	−.380**	−.396**	−.373**	−.434**
	Personal accomplishment	−.260**	−.298**	−.283**	−.279**	−.275**	−.329**

JSP-HE = Jefferson Scale of Empathy-Health Professionals, MBI-GS = Maslach Burnout Inventory-General Survey, PCC = person-centered care, PCPON = the Person-Centered Perioperative Nursing Scale.

** Correlation is significant at the 0.01 level (2-tailed).

### 3.4. Factors influencing surgical nurses’ PCC competence

Multiple regression analysis was performed to identify the factors influencing surgical nurses’ PCC competence. Table 4 presents the results of this study.

**Table 4:** Multiple regression analysis of PCC competence with the variables (n = 437).

Variables	PCC competence				
	B	SE	*β*	t	*P*-value
(Constant)	53.115	3.388		15.675	<.001
Empathy	0.310	0.022	0.502	13.821	<.001
Job burnout	−0.224	0.028	−0.288	−8.067	<.001
Nursing humanistic training	4.582	0.877	0.167	5.225	<.001
Personnel agency	1.830	0.679	0.083	2.697	.007
F (P)	158.930				<.001
*R^2^*	0.595				
Adjusted *R*^2^	0.592				

Durbin–Watson = 0.720.

Multiple regression analysis was performed for all general characteristics, including age, level of education, marital status, professional title, duration of work as a nurse, place of residence, employment status, humanistic nursing training, empathy, and burnout. Homogeneity, multicollinearity of residuals, and normal distributions were examined. The nominal scale variable was selected as a dummy variable for all the predictor variables. The tolerance ranged from 0.711 to 0.978 (above 0.1) and the variation inflation factor ranged from 1.022 to 1.406 (below 10), confirming that there was no risk of multicollinearity among the independent quantities. The Durbin–Watson residual independence test statistic was 0.720, which is commonly used for vertical data analyses. This study uses the data of a cross-sectional questionnaire study, whereas the level of this statistic has little effect on autocorrelation among the error terms (Wu, 2010, p. 387).

The regression model for surgical nurses’ PCC competence was significant (*F* = 158.930, *P* < .001) indicating that PCC competence was influenced by empathy (*β* **= **0.502, *P* < .001), job burnout (*β* = −0.288, *P* < .001), humanistic nursing training (*β* **= **0.167, *P* < .001), and personnel agency (*β* **= **0.083, *P* < .001). All 4 contributing factors accounted for 59.2% of the variation. In this study, age, marital status, educational level, professional title, and other variables did not significantly affect nursing ability.

## 4. Discussion

The main finding of our study was the factors promoting PCC competence among clinical and surgical nurses in China as empathy, job burnout, humanistic nursing training, and personnel agency (employment status) during the perioperative period. The results of our study showed that surgical nurses’ PCC competence was above the medium level. Also, there was a positive relationship between nursing competence and levels of empathy, and that job burnout may be a negative predictor of humanistic abilities. These meaningful conclusions may contribute to a better understanding of the factors that improve the ability to strengthen this crucial skill in nursing practice.

It has been demonstrated that empathy was the most critical factor, and participants with high empathy had higher PCC competence. Empathy, as a comprehensive, multi-component term with both affective and cognitive components has been reported to influence the person-centered attitudes of healthcare providers.^[12]^ It has been a crucial contributor in communicating the patient’s emotions and concerns, which can help them be considered as decision-making partners.^[25]^ Similar observations have confirmed that empathy is appropriate for predicting and describing humanistic care ability among undergraduate nursing students.^[12,15]^ The JSP-HE was used in our study because it is well-adapted to measure surgical nurses’ empathy in various clinical situations. The level of empathy measured was slightly higher than that of other research data in 2018, possibly because of the different subjects studied.^[26]^ However, compassionate care, which belongs to the subdimension of empathy, was lower than that of Chinese nurses in 2018.^[26]^ Empathy is also considered one of the foundation components of therapeutic nurse–patient relationship.^[27]^ Patient needs and concerns can be realized through effective communication. High levels of empathy help reduce healthcare risks, conflicts, and disputes and improve patient well-being.^[26,28]^ Ongoing practical nursing skills are required to ensure that empathy is prominent and well developed, as nurses must play a key role in meeting patients’ needs through personalized access rather than just focusing on urgent care. According to the findings of our study, compassionate interaction, respect, comfort, information sharing, and expertise in PCC competency are all positively associated with perspective-taking, compassionate care, and walking in patients’ shoes. Therefore, it is helpful for surgical nurses to increase their empathy level to achieve high-quality competence.

Job burnout was highly significant and negatively correlated with PCC competence in our study, and was an influential determinant in surgical nurses. The term “burnout” was first defined clinically by Maslach as a state of exhaustion and depletion caused by excessive demands on energy, resources or strength, usually in the case of professional workers.^[29]^ Surgical nurses, as a service group, work long hours under pressure, high workloads, complex relationships, and inappropriate coping strategies lead to burnout. Burnout is a psychological syndrome that includes emotional exhaustion, depersonalization and personal accomplishment, affects nurses’ physical and mental health, and is closely linked to nursing quality.^[30]^ Empathy is a moral choice, and compassionate nurses can overcome burnout and provide quality care to patients. Hospital demands and expectations of nurses have increased due to the rapid expansion of China’s medical model, particularly the Plan for a Healthy China 2030, to raise the quality of nursing service. It engenders transformations within the nursing profession and readily contributes to physical and mental exhaustion, thereby augmenting the susceptibility of nurses to burnout. All these factors directly lead to a decline in nurses’ compassionate care. PCC competencies were adversely related to burnout scores, and PCC competencies increased as burnout declined. Furthermore, surgical nurses’ overall burnout scores were similar to those of primary care practitioners in China. Still, surgical nurses showed greater burnout in the depersonalization dimension, where individuals ignored the personality of the person they were caring for.^[31]^ These results suggest that burnout may be a serious disadvantage in the performance of PCC.

In addition, our study also showed a significant connection between PCC proficiency and humanistic training. Hospital culture is the long-term development of values and behaviors agreed upon and followed by all staff members. Teaching healthcare providers empathy and person-centered care lies at the heart of humanistic medical education.^[30]^ First, nursing is a humanistic profession that requires technical, medical, and communicative competence.^[32]^ The original intention of every nurse was to protect their lives and treat casualties. Medical professional ethics full of humanistic spirit are their moral standard in daily service work.^[33]^ Since commencing work, 85.1% of surgical nurses have undergone more than 1 hour of humanistic nursing training. Humanistic nursing training has become essential for clinical nursing activities, designed to integrate humanistic principles. Similarly, Scerri et al found that care competency frameworks for nurses recommend developing training programs with competencies ranging from intellectual and interpersonal to attitudinal skills to improve the quality of care for chronic diseases such as dementia.^[34]^ To develop empathetic medical care personnel, an important PCC initiative incorporating empathy and humanistic values into educational programs was recently launched at the University of Toronto Faculty of Medicine.^[30]^ Furthermore, Stanhope et al observed that the experimental condition received training in PCC by providing educational materials such as manuals and training slides.^[35]^ In contrast, the control condition delivered service planning based on problem-focused care. These results demonstrate that training practitioners is a good way to strengthen humanistic ability delivery.^[35]^ In recent years, the concept of humanistic spirit and the content of humanistic nursing training have been debated, but there is still no clear definition of these terms. Bai J proposed that a humanitarian spirit must consist of caring for life, saving the injured, having a sense of morality and responsibility, and human kindness.^[33]^ The strained relationship between nurses and patients in medical institutions can be effectively relieved by humanistic nursing training that includes the above mentioned concepts, which have been a major driver of humanistic medical education in the clinical environment.

The fourth factor was personnel agency as employment status related to nursing competence. The level of employment determines the professional mentality of nurses and influences the quality of humanistic care they provide. In China’s nursing management system, employment status is more linked to nurses’ salaries and personnel evaluation, and personnel agents’ wages are higher than those of ordinary labor dispatchers. Personnel agency nurses tend to have higher levels of education and stronger professional skills than contract nurses, and they will have more advantages in terms of professional advancement, management, and other rights, which helps them develop their careers and feel more satisfied and valued. It was found that the number of personnel agencies and authorized personnel nurses is much lower than that of labor-dispatching nurses and that they have higher levels of PCC competence than the latter in our study. As a human resource management system, personnel agencies are more stable and long-lasting than labor dispatching. Many hospitals have introduced flexible human resource management systems to improve staff motivation and efficiency.^[36]^ However, previous studies have not shown a link between the type of nurses employed and their ability to provide humanistic nursing, indicating that more research is needed. In addition, as shown here, gender and belonging to an only child family do not influence PCC competence. Similar results were reported in a study by Ahn and Yi.^[16]^

The quality of perioperative nursing is related to the safety, satisfaction, and health of the patient which is the core content of nursing. In our research, we considered PCC competence as an indicator that best represents the humanistic quality of nursing through personal subjective judgment rather than the influence of an objective setting. There has been a gradual increase in recent research in which PCC competence has been used as the outcome variable. However, there is a shortage of studies on nurses’ ability, particularly in the surgical environment. Our study fills a gap in this field, at least in China, and helps us put our research on humanistic care at the core. In addition, several policy choices and individual efforts are essential to improving humanistic literacy.

This study identified factors that influence surgical nurses’ perioperative human-centered nursing skills. A high standard of patient care is directly linked to these competencies. Therefore, the sense of patients’ hospitalization experience and the image of medical institutions can be improved by improving these abilities. Concerning nursing practice, this study identifies nurses’ empathy and burnout levels, whether they receive humanistic nursing training, employment status, and PCC competence, thus providing reference variables for clinical interventions.

## 5. Limitations

Our study has some limitations. First, our sampling strategy was based on recruiting surgical nurses from tertiary hospitals in Anhui, China, and its representativeness needs to be confirmed. Second, the survey was conducted through an online questionnaire. The participants were informed before the research. Nonetheless, the study’s confidentiality and anonymity measures imposed restrictions on participants’ responses, primarily due to the influence of social expectation bias. Third, as the nursing profession has developed, the number of males in the nursing profession has steadily increased. In contrast, most participants in our study were female, thus limiting the representativeness and generalizability of the findings.

## 6. Conclusions

The present results demonstrate that the factors influencing PCC include empathy, job burnout, humanistic nursing training, and employment status. Despite certain limitations, the results suggest that treatments to promote PCC among surgical nurses include promoting empathy, decreasing burnout, strengthening human-centered nursing education, and improving staff treatment and work circumstances. Consequently, future research should explore factors affecting competence in humanistic nursing practice.

## Acknowledgments

The authors would like to acknowledge the 452 registered nurses for their participation and support.

## Author contributions

**Conceptualization:** Lu Shen.

**Data curation:** Lu Shen, Dongju Xu.

**Investigation:** Lu Shen, Dongju Xu.

**Methodology:** Lu Shen, Xiumei Zhang.

**Resources:** Xiumei Zhang.

**Software:** Lu Shen.

**Supervision:** Xiumei Zhang.

**Validation:** Dongju Xu.

**Writing – original draft:** Lu Shen.

**Writing – review & editing:** Xiumei Zhang.

## References

[1] Dana F Sebio-García R Tena B. Perioperative nursing as the guiding thread of a prehabilitation program. Cancers. 2022;14:5376. 36358794 10.3390/cancers14215376 PMC9653559

[2] Alanazi NH Alshamlani Y Baker OG. The association between nurse managers’ transformational leadership and quality of patient care: a systematic review. Int Nurs Rev. 2023;70:175–84. 36583960 10.1111/inr.12819

[3] Håkansson Eklund J Holmström IK Kumlin T. “Same same or different?” A review of reviews of person-centered and patient-centered care. Patient Educ Couns. 2019;102:3–11. 30201221 10.1016/j.pec.2018.08.029

[4] McCormack B McCance TV. Development of a framework for person-centred nursing. J Adv Nurs. 2006;56:472–9. 17078823 10.1111/j.1365-2648.2006.04042.x

[5] McCormack B. Person-centredness and fundamentals of care - dancing with beauty rather than fighting ugliness. Nurs leadersh(Tor Ont). 2016;29:17–25. 27309638 10.12927/cjnl.2016.24642

[6] Liang Z Xu M Liu G Zhou Y Howard P. Patient-centred care and patient autonomy: doctors’ views in Chinese hospitals. BMC Med Ethics. 2022;23:38. 35395761 10.1186/s12910-022-00777-w PMC8994393

[7] Alrasheadi BA Alamri MS Aljohani KA. nurses’ perception of safety culture in medical−surgical units in hospitals in Saudi Arabia. Medicina. 2022;58:897. 35888615 10.3390/medicina58070897 PMC9321783

[8] Armstrong BA Dutescu IA Nemoy L. Effect of the surgical safety checklist on provider and patient outcomes: a systematic review. BMJ QUAL SAF. 2022;31:463–78. 10.1136/bmjqs-2021-014361 35393355

[9] Gethin G Probst S Stryja J Christiansen N Price P. Evidence for person-centred care in chronic wound care: a systematic review and recommendations for practice. J Wound Care. 2020;29:S1–S22. 10.12968/jowc.2020.29.Sup9b.S1 32935648

[10] Rajamohan S Porock D Chang YP. Understanding the relationship between staff and job satisfaction, stress, turnover, and staff outcomes in the person-centered care nursing home arena. J Nurs Scholarsh. 2019;51:560–8. 31245922 10.1111/jnu.12488

[11] Ahn J Kim M. Influencing factors on person-centered care competence among nursing students experienced clinical training. Medicina. 2021;57:1295. 34946240 10.3390/medicina57121295 PMC8704963

[12] Park E Choi J. Attributes associated with person-centered care competence among undergraduate nursing students. Res Nurs Health. 2020;43:511–9. 32780468 10.1002/nur.22062

[13] Kim M. The factors associated with person-centered care competence among nursing students. Int J Environ Res Public Health. 2022;19:2787. 35270478 10.3390/ijerph19052787 PMC8910480

[14] Jeon J Choi S. Factors influencing patient-centeredness among korean nursing students: empathy and communication self-efficacy. Healthcare (Basel, Switzerland). 2021;9:727. 34204788 10.3390/healthcare9060727 PMC8231486

[15] Yun J Cho I. Structural equation model for developing person-centered care competency among senior nursing students. Int J Environ Res Public Health. 2021;18:10421. 34639721 10.3390/ijerph181910421 PMC8508033

[16] Ahn S Yi Y. Factors influencing mental health nurses in providing person-centered care. Nurs Ethics. 2022;29:1491–502. 35723256 10.1177/09697330221089076

[17] Aspden T Egan JP Bye L Petersen L. Using visual thinking strategies to support development of pharmacy student competency in person-centered care. Am J Pharm Educ. 2022;86:8607. 34385170 10.5688/ajpe8607 PMC10159445

[18] Al-Sahli B Eldali A Aljuaid M Al-Surimi K. Person-centered care in a tertiary hospital through patient’s eyes: a cross-sectional study. Patient Prefer Adherence. 2021;15:761–73. 33883884 10.2147/PPA.S286237 PMC8055245

[19] Roen I Kirkevold O Testad I Selbaek G Engedal K Bergh S. Person-centered care in Norwegian nursing homes and its relation to organizational factors and staff characteristics: a cross-sectional survey. Int Psychogeriatr. 2018;30:1279–90. 29198221 10.1017/S1041610217002708 PMC6190067

[20] Shin S Kang J. Development and validation of a person-centered perioperative nursing scale. Asian Nurs Res. 2019;13:221–7. 10.1016/j.anr.2019.07.002 31351143

[21] Lu S Huiping Li Hui W. Chinese version of person⁃centered perioperative nursing scale and its relicompetence and validity test. Chin Nurs Res. 2021;35:1904–8. in Chinese.

[22] Xiuqin A Hui Y Jianping X. complication and evaluation of Jefferson empathy scaIe. Chin Nurs Res. 2008;22:2063–6. in Chinese.

[23] Chaoping L kan S. The influence of distributive justice and procedural Justice on job burnout. Psychol Sci. 2003;35:677-–84. in Chinese.

[24] Yongxin Li Yimin Li A preliminary discussion on the evaluation criteria of job Burnout. Psychol Sci. 2006;29:148–50. in Chinese.

[25] Lee M Ihm J. Empathy and attitude toward communication skill learning as a predictor of patient-centered attitude: a cross-sectional study of dental students in Korea. BMC Med Educ. 2021;21:225. 33882935 10.1186/s12909-021-02674-z PMC8058758

[26] Yi X Sicheng X Lihui Z. Changes in empathy of nurses from 2009 to 2018: a cross-temporal meta-analysis. Nurs Ethics. 2021;28:776–90. 33283617 10.1177/0969733020968163

[27] Moreno-Poyato AR Rodriguez-Nogueira O. The association between empathy and the nurse-patient therapeutic relationship in mental health units: a cross-sectional study. J Psychiatr Ment Health Nurs. 2021;28:335–43. 32657511 10.1111/jpm.12675

[28] Du J Huang S Lu Q Ma L Lai K Li K. Influence of empathy and professional values on ethical decision-making of emergency nurses: a cross sectional study. Int Emerg Nurs. 2022;63:101186. 35749969 10.1016/j.ienj.2022.101186

[29] Ostacoli L Cavallo M Zuffranieri M. Comparison of experienced burnout symptoms in specialist oncology nurses working in hospital oncology units or in hospices. Palliat Support Care. 2010;8:427–32. 20875206 10.1017/S1478951510000295

[30] Kuper A Boyd VA Veinot P. A dialogic approach to teaching person-centered care in graduate medical education. J Grad Med Educ. 2019;11:460–7. 31440342 10.4300/JGME-D-19-00085.1 PMC6699535

[31] Jing YR Han WT Qin WZ. Burnout and associated factors among family doctor team members in different types of primary healthcare institutions: a comparative study. Chin General Pract. 2022;25:829–836, 845. in Chinese.

[32] Purssell E McCrae N. The rise and fall of university-based nurse training. Nurse Educ Pract. 2021;56:103081. 34600409 10.1016/j.nepr.2021.103081

[33] Bai J. Humanistic spirit training of medical students based on multisource medical data fusion. Comput Math Methods Med. 2022;2022:7896367. 35936381 10.1155/2022/7896367 PMC9355777

[34] Scerri A Innes A Scerri C. Person-centered dementia care in acute hospital wards-The influence of staff knowledge and attitudes. Geriatr Nurs. 2020;41:215–21. 31630871 10.1016/j.gerinurse.2019.09.001

[35] Stanhope V Choy-Brown M Williams N Marcus SC. Implementing person-centered care planning: a randomized controlled trial. Psychiatric Services (Washington, D.C.). 2021;72:641–6. 33765860 10.1176/appi.ps.202000361 PMC8192424

[36] Costa C Vedovetto A Tasca T. The resignations among nurses in the Veneto healthcare institutions. A retrospective study. Assist Inferm Ric. 2023;42:60–72. 37309657 10.1702/4050.40312

